# Physical activity during and after breast cancer therapy and associations of baseline physical activity with changes in cardiac function by echocardiography

**DOI:** 10.1002/cam4.3277

**Published:** 2020-07-09

**Authors:** Jenica N. Upshaw, Rebecca A. Hubbard, Jian Hu, Justin C. Brown, Amanda M. Smith, Biniyam Demissei, Kathryn H. Schmitz, Bonnie Ky

**Affiliations:** ^1^ Department of Medicine Division of Cardiology Tufts Medical Center Boston MA USA; ^2^ Department of Biostatistics, Epidemiology & Informatics Perelman School of Medicine at the University of Pennsylvania Philadelphia PA USA; ^3^ Department of Population and Public Health Sciences Pennington Biomedical Research Center Baton Rouge LA USA; ^4^ Department of Public Health Sciences Pennsylvania State College of Medicine Hershey PA USA; ^5^ Department of Medicine Division of Cardiology Perelman School of Medicine at the University of Pennsylvania Philadelphia PA USA

**Keywords:** anthracyclines, breast cancer, chemotherapy, exercise, trastuzumab

## Abstract

**Background:**

Observational studies suggest that regular physical activity may reduce cardiovascular morbidity and cancer recurrence in survivors of breast cancer. The association between physical activity and cardiac function with breast cancer therapy is unknown.

**Methods:**

Self‐reported physical activity was assessed using the Godin Leisure‐Time Exercise Questionnaire at repeated intervals in a longitudinal cohort study of 603 breast cancer participants treated with doxorubicin and/or trastuzumab. Multivariable regression models estimated associations between clinical variables and physical activity. Generalized estimating equations adjusted for prespecified variables estimated associations between baseline physical activity and longitudinal echocardiographic measures of systolic and diastolic function.

**Results:**

Physical activity was low at baseline, prior to cancer therapy initiation. More than half of participants reported no moderate‐strenuous physical activity, and only 12.1% met guideline recommended levels of physical activity. Physical activity increased after chemotherapy completion; however, only 26.0% of individuals were sufficiently active 3 years after cancer diagnosis. Body mass index, hyperlipidemia and higher cancer stage were significantly associated with lower total physical activity at baseline. Higher baseline physical activity was very modestly associated with an attenuation in the absolute decline in left ventricular ejection fraction over time (β 0.4%, 95% CI 0.1, 0.7, *P* = .02 for each 10‐unit increase in total activity). There tended to be a cardioprotective association with cardiotoxicity risk, although this was not statistically significant (HR 0.83, 95% CI 0.66, 1.04, *P* = .097).

**Conclusions:**

A small proportion of breast cancer patients and survivors are engaged in regular moderate‐strenuous physical activity. While only modest associations between self‐reported physical activity and left ventricular systolic function were observed, our findings do not exclude a cardioprotective benefit of exercise.

## Graphical abstract

Self‐reported physical activity was assessed using the Godin Leisure‐Time Exercise Questionnaire at repeated intervals in a longitudinal cohort study of 603 breast cancer participants treated with doxorubicin and/or trastuzumab. Physical activity was low at baseline and increased somewhat after chemotherapy completion however, only 26.0% of individuals were sufficiently active 3 years after cancer diagnosis. Higher baseline physical activity was very modestly associated with an attenuation in the absolute decline in left ventricular ejection fraction over time.

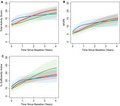

## 1 INTRODUCTION

Cardiovascular disease has emerged as a major cause of long‐term noncancer related morbidity and mortality in patients with breast cancer due to reductions in breast cancer mortality and the cardiovascular toxicity of breast cancer treatment.^1^, ^2^ Anthracyclines, such as doxorubicin, cause oxidative and nitrosative stress, mitochondrial dysfunction, and cardiomyocyte apoptosis, resulting in reductions in left ventricular ejection fraction (LVEF) and heart failure (HF).^1^, ^3^ Monoclonal antibodies that block ErbB2 (HER2/neu) signaling, such as trastuzumab, interfere with cardiac homeostasis and can also result in LVEF reductions and HF.^2^ Chest radiation therapy with incidental dose to cardiac substructures can lead to accelerated atherosclerosis, myocardial fibrosis, ischemic heart disease and heart failure.^4^ Patients with breast cancer often receive combinations of these potentially cardiotoxic therapies, and thus are at increased risk for cardiovascular disease.

In the general population, there is a dose‐dependent relationship between greater self‐reported regular physical activity and reduced risk of cardiovascular events and incident cancer.^5^ Similarly, in those with established coronary artery disease or heart failure, regular physical activity is associated with improved outcomes.^6^ In breast cancer cohort studies, higher self‐reported physical activity prior to, during, or after cancer treatment has been associated with reduced risk of cancer and all‐cause mortality,^7^ and a reduced cardiovascular events.^8^

There are limited prospective data on the associations between self‐reported physical activity and changes in echocardiographic measures of systolic and diastolic function with breast cancer therapy.^9^ In this cohort, we had previously defined both the changes in left ventricular systolic and diastolic function with exposure to cancer therapy, which are on the order of LVEF −6.6% (95% CI, −8.2, −5.0%), longitudinal strain 0.9% (95% CI, 0.3, 1.5%), E/e′ 0.8 (0.3, 1.4) for sequential doxorubicin and trastuzumab.^3^, ^10^ Here, we performed a detailed analysis of the changes in self‐reported physical activity in a well‐phenotyped cohort of 603 participants with breast cancer undergoing doxorubicin and/or trastuzumab therapy to determine: (a) changes in self‐reported physical activity with cancer treatment; (b) baseline predictors of physical activity during and after cancer treatment; and (c) the associations between baseline self‐reported physical activity and changes in echocardiography‐derived parameters of systolic and diastolic function after cancer therapy. For the latter, we focused solely on baseline physical activity given concerns over reverse causation.^11^

## 2 METHODS

### 2.1 Study population

The Cardiotoxicity of Cancer Therapy (CCT) study is a longitudinal prospective cohort study of breast cancer participants undergoing treatment with doxorubicin and/or trastuzumab therapy at the Rena Rowan Breast Cancer Center at the University of Pennsylvania (NCT01173341). The study protocol has been previously described (Figure S1).^3^ Briefly, participants at least 18 years of age with a diagnosis of breast cancer with planned treatment with doxorubicin and/or trastuzumab were eligible to participate. The only exclusion criteria were pregnancy and inability to provide informed consent. The baseline study visit occurred soon after breast cancer diagnosis but immediately before initiation of systemic cancer therapy. The study was approved by the Institutional Review Board at the University of Pennsylvania, and all participants provided written informed consent.

### 2.2 Cancer treatment and clinical characteristics

Breast cancer treatment was determined by the treating oncologist and for the purposes of this study classified into three categories: (a) doxorubicin (240 mg/m^2^) with concurrent cyclophosphamide, followed by paclitaxel (Dox); (b) trastuzumab with docetaxel and either cyclophosphamide or carboplatin (Tras); or (c) doxorubicin (240 mg/m^2^) with concurrent cyclophosphamide, followed by trastuzumab and paclitaxel (Dox+Tras). Exposure to additional cardiotoxic therapies, including pertuzumab and radiation were also noted. Cancer‐related clinical variables such as clinical stage, hormone receptor status and surgical therapy were collected by clinical chart review. Clinical characteristics such as hypertension, diabetes, hyperlipidemia, tobacco use, and body mass index (BMI) were assessed by participant report, clinical chart review, and review of prescribed medications.

### 2.3 Self‐reported physical activity

Physical activity was assessed using the validated Godin Leisure‐Time Exercise Questionnaire (Godin),^12^ a self‐administered questionnaire that asks the number of times one engages in mild (minimal effort), moderate (not exhausting) and strenuous (heart beats rapidly) leisure time physical activity of at least 15 minutes in duration in a typical 7 day period (Supplemental Methods). We derived three variables of interest from the Godin questionnaire: (a) Total Physical Activity Score (Total Activity), defined as the number of activity sessions in a given 7 day period multiplied by the approximate MET value (unitless sum of strenuous activity multiplied by 9, moderate activity by 5 and mild activity by 3); (b) Moderate‐Strenuous Physical Activity Score (Moderate‐Strenuous Activity), which only included moderate and strenuous activity; (c) Sufficiently Active, a binary variable defined as a moderate‐strenuous activity summary score of 24 or greater, which approximates the national recommendation of at least 150 minutes of moderate‐strenuous physical activity per week.^12^ Participants were asked to complete the questionnaires at the baseline visit—typically the same day that cancer therapy was initiated, then every 4‐6 weeks during cancer therapy, and annually after the completion of cancer therapy (Table S1).

### 2.4 Echocardiographic quantitation and outcomes

Transthoracic echocardiograms were performed by dedicated sonographers at an Intersocietal Accreditation Commission laboratory using primarily GE Vivid E7, E9, or E95 machines (GE Healthcare).^3^ Echocardiograms were obtained at baseline in all participants, at standardized intervals during treatment, and annually (Figure S1). Echocardiography images were archived, with blinded core‐lab quantitation of systolic function parameters, including Simpson's derived LVEF and longitudinal strain, and diastolic function parameters, including E/e′ and diastolic function grade (TomTec Imaging Systems).^13^ Cancer therapeutics‐related cardiac dysfunction (CTRCD) was defined as a quantitated LVEF decline of ≥10% to a value of <50%.

### 2.5 Statistical analysis

Baseline characteristics were summarized according to treatment regimen (Dox, Tras, Dox + Tras) using median (IQR) for continuous variables and proportions for categorical variables. The mean change over time in the total activity score, moderate‐vigorous activity score and proportion sufficiently active at 6, 12, 24, and 36 months since initiation of cancer therapy for each regimen was estimated using linear regression estimated via generalized estimating equations (GEE). These models were adjusted for time since initiation of cancer therapy and the interaction of time with treatment regimen. A robust variance estimator was used to account for clustering within subjects, and time since initiation of cancer therapy was modeled using a cubic spline with 3 *degrees of freedom (df)*. Hypothesis tests for change in each parameter at each time point relative to baseline were conducted using Wald tests.

Multivariable linear regression models estimated via GEE were used to estimate the association between baseline demographic and clinical variables and the baseline physical activity score as well as rates of change in physical activity scores over time (one model included baseline and rates of change overtime). Logistic regression models estimated these associations for the outcome of proportion sufficiently active. Main effects from these models represent adjusted differences in average baseline activity while interaction parameters represent differences in the average rate of change in activity as a function of covariates. Models included time, treatment, baseline covariates (age, race, BMI, hypertension, smoking status, hyperlipidemia, beta blocker use, and cancer stage at diagnosis), and interactions between each baseline covariate and time. The longitudinal change over time in self‐reported total and moderate‐strenuous activity scores as well as percent sufficiently active was also assessed graphically according to treatment arm using natural cubic splines with 3 *df*.

The association between baseline activity measures (total activity score, moderate‐vigorous activity score, sufficiently active) and changes in LVEF, longitudinal stratin, and E/e′ were estimated using a GEE model adjusted for prespecified variables (treatment, age, race, cancer stage, BMI, hypertension, and hyperlipidemia) and the time since baseline modeled as a natural cubic spline with 3 *df*. All patients with a quantitated baseline echocardiogram and at least one quantitated follow‐up echocardiogram were included in these analyses. These analyses were repeated with the outcomes of time to CTRCD and incident abnormal diastolic function grade using Cox proportional hazards models. Here, we focused only on associations with baseline physical activity, given concerns over reverse causation.^11^

## 3 RESULTS

### 3.1 Study population

In 603 participants followed for a median of 2.3 years (IQR 1.0, 4.4), physical activity surveys were collected at standardized intervals (median of 7 [IQR 4, 10] surveys per participant; Table S1). Baseline demographic, clinical, and echocardiographic characteristics are summarized in Table 1. The median age was 50 years, 70% were Caucasian and the median body mass index (BMI) was 26 kg/m^2^. Cardiac risk factors including hypertension, hyperlipidemia, diabetes, and current/former tobacco use were common, occurring in 30%, 24%, 9%, and 35%, respectively.

**Table 1:** Baseline demographic, clinical and echocardiographic variables stratified according to cancer treatment

	Overall (N = 603)	Doxorubicin (N = 360)	Trastuzumab (N = 156)	Doxorubicin + Trastuzumab (N = 87)
Age (y), median (IQR)	50 (42, 58)	50 (42, 57)	52 (44, 60)	45 (38, 55)
Race, N (%)				
Caucasian	424 (70.4)	245 (68.2)	122 (78.2)	57 (65.5)
African American	152 (25.2)	104 (29.0)	24 (15.4)	24 (27.6)
Other	26 (4.3)	10 (2.8)	10 (6.4)	6 (6.9)
Hispanic	14 (2.3)	9 (2.5)	2 (1.3)	3 (3.4)
BMI (kg/m^2^), median (IQR)	26.4 (23.1, 31.6)	26.7 (23.1, 31.9)	26.0 (23.3, 31.4)	26.5 (22.7, 30.9)
SBP (mm Hg), median (IQR)	125 (115, 136)	125 (115, 136)	127 (117, 141)	124 (112, 133)
DBP (mm Hg), median (IQR)	76 (70, 82)	76 (70, 83)	75 (70, 81)	74 (70, 81)
Heart rate, median (IQR)	78 (70, 88)	79 (70, 88)	76 (68, 88)	79 (73, 87)
Hypertension, N (%)	181 (30.1)	112 (31.2)	53 (34.0)	16 (18.4)
Diabetes, N (%)	54 (9.0)	36 (10.1)	13 (8.4)	5 (5.8)
Hyperlipidemia, N (%)	142 (23.7)	83 (23.2)	40 (25.8)	19 (21.8)
Tobacco use, current or former, N (%)	198 (35.4)	129 (38.5)	44 (30.3)	25 (31.2)
Angiotensin receptor blocker, N (%)	41 (6.8)	26 (7.2)	14 (9.0)	1 (1.1)
ACE inhibitor, N (%)	49 (8.1)	34 (9.4)	11 (7.1)	4 (4.6)
Beta blocker, N (%)	53 (8.8)	33 (9.2)	15 (9.6)	5 (5.7)
Calcium channel blocker, N (%)	55 (9.1)	30 (8.3)	16 (10.3)	9 (10.3)
Diuretics, N (%)	78 (12.9)	52 (14.4)	20 (12.8)	6 (6.9)
Disease site, N (%)				
Bilateral	25 (4.2)	17 (4.7)	2 (1.3)	6 (7.0)
Left‐sided	282 (46.9)	182 (50.6)	63 (40.6)	37 (43.0)
Right‐sided	284 (47.3)	157 (43.6)	84 (54.2)	43 (50.0)
Stage, N (%)				
1	132 (22.0)	55 (15.4)	64 (41.0)	13 (15.3)
2	325 (54.3)	211 (58.9)	65 (41.7)	49 (57.6)
3	133 (22.2)	90 (25.1)	20 (12.8)	23 (27.1)
4	9 (1.5)	2 (0.6)	7 (4.5)	0 (0.0)
Radiation therapy, N (%)	373 (64.9)	231 (66.8)	86 (58.9)	56 (67.5)
LVEF, Median (IQR)	53.8 (51.1, 56.7)	53.5 (50.5, 55.8)	53.3 (51.8, 56.5)	55.2 (53.2, 58.1)
E/e' average, Median (IQR)	6.9 (5.6, 8.6)	6.9 (5.4, 8.4)	7.2 (6.1, 9.3)	6.5 (5.7, 7.7)
Abnormal diastolic grade, N (%)	75 (20.7)	47 (21.4)	16 (20.5)	12 (18.8)
Total activity score, Median (IQR)	9.0 (0.0, 21.0)	8.2 (0.0, 21.0)	10.2 (0.0, 21.0)	9.0 (0.0, 21.0)
Moderate‐strenuous activity score, Median (IQR)	0.0 (0.0, 5.0)	0.0 (0.0, 5.0)	0.0 (0.0, 10.0)	0.0 (0.0, 4.5)
Sufficiently active, N (%)	73 (12.1)	44 (12.2)	17 (10.9)	12 (13.8)

Abbreviations: BMI, body mass index; DBP, diastolic blood pressure; E, early mitral inflow peak velocity (cm/sec); e′, pulse wave tissue Doppler of the lateral and septal annulus; LVEF, left ventricular ejection fraction; SBP, systolic blood pressure.

Sixty percent of the participants received doxorubicin without trastuzumab (Dox), 26% received trastuzumab without doxorubicin (Tras) and 14% received doxorubicin followed by trastuzumab (Dox + Tras). Sixty‐five percent of participants also received radiation therapy.

### 3.2 Baseline self‐reported physical activity

At baseline, the self‐reported total leisure‐time activity score was low with a median score of 9 (IQR 0.21) and median moderate‐vigorous activity score of 0 (IQR 0.5; Table 1). Only 12.1% of participants at baseline were sufficiently active. In multivariable analysis, higher BMI, hyperlipidemia and higher cancer stage at diagnosis were associated with a lower total baseline activity score (Table 2). Similar associations with BMI, hyperlipidemia and cancer stage were observed for the outcomes of moderate‐strenuous activity and the odds of being sufficiently active (Tables S2 and S3).

**Table 2:** Associations between baseline clinical characteristics and baseline total activity score and longitudinal rate of change in total activity score

	Baseline difference	95% CI	*P*‐value	Difference in rate of change	95% CI	*P*‐value
Treatment (reference Dox)						
Tras	1.53	(−0.87, 3.93)	.211	−0.86	(−2.00, 0.29)	.144
Dox + Tras	−1.20	(−3.64, 1.24)	.334	0.12	(−0.82, 1.06)	.802
Age at baseline, quartiles, (Reference < 41 y)						
Quartile 2:42‐49 y	−0.41	(−3.18, 2.36)	.769	−0.17	(−1.59, 1.25)	.813
Quartile 3:50‐58 y	2.92	(−0.33, 6.17)	.079	−0.84	(−2.44, 0.77)	.309
Quartile 4: >58 y	2.49	(−0.72, 5.71)	.129	−1.03	(−2.61, 0.55)	.202
Race (reference Caucasian)						
African American	0.30	(−2.06, 2.66)	.804	−0.99	(−2.07, 0.09)	.072
Other	−1.70	(−5.39, 2.00)	.369	−1.73	(−3.26, −0.20)	.026
Body mass index	−0.22	(−0.38, −0.06)	.008	−0.09	(−0.17, −0.01)	.028
Hypertension	−2.13	(−4.48, 0.21)	.075	−0.29	(−1.25, 0.67)	.549
Smoking	−1.01	(−3.03, 1.01)	.326	0.71	(−0.27, 1.70)	.155
Hyperlipidemia	−2.81	(−5.04, −0.59)	.013	−0.17	(−1.12, 0.77)	.719
Beta blockers	−1.51	(−4.36, 1.34)	.300	0.78	(−0.66, 2.22)	.290
Stage (reference stage 1)						
2	−1.78	(−4.66, 1.10)	.225	0.14	(−0.90, 1.18)	.793
3	−3.58	(−6.58, −0.57)	.020	0.15	(−1.21, 1.51)	.826
4	−4.89	(−10.50, 0.72)	.088	−1.09	(−3.33, 1.15)	.340

Note

Multivariable linear regression model used to estimate the association between all listed baseline demographic and clinical variables and difference in the baseline total activity scores and the difference in the rate of change in the activity score during longitudinal follow‐up.

Abbreviations: Dox, doxorubicin; Tras, trastuzumab.

### 3.3 Changes in self‐reported physical activity during and after cancer treatment

**Figure 1:**
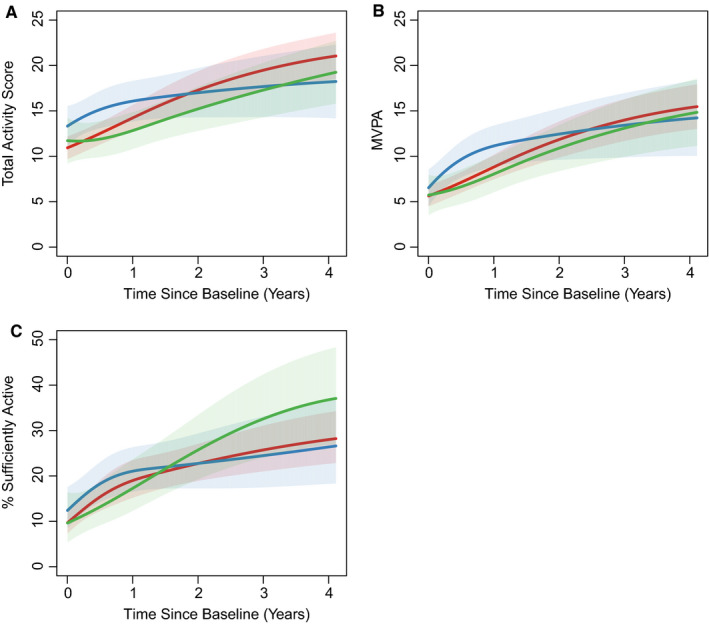
Spline of total activity score (A), Moderate vigorous physical activity score (B), and percent sufficiently active (C) with 95% confidence intervals stratified by treatment regimen: doxorubicin (red), trastuzumab (blue), doxorubicin and trastuzumab (green)

In participants treated with Dox, there was a modest, but nonsignificant initial decrease in self‐reported physical activity followed by an increase in physical activity, such that by 12 months after treatment initiation, the mean total activity scores were on average 2.21 points higher (95% CI 0.72, 3.71) than baseline and continued to increase to a mean 6.95 points higher (95% CI 4.40, 9.50) 36 months after beginning treatment (Table 3; Figure 1). In Dox + Tras treated participants the same pattern of an early decrease followed by an increase after completion of treatment was seen but this increase was delayed. At 24 months there was a mean 2.96 point increase (95% CI 0.23, 5.69) and by 36 months there was a mean 5.33 point increase in total activity score compared with baseline (95% CI 2.04, 8.63). In Tras‐treated patients there was no initial decrease. The total activity score increased during and after treatment with mean scores that were 3.76 points higher (95% CI 0.97, 6.55) at 24 months and 5.11 points higher (95% CI 1.87, 8.34) at 36 months after baseline assessment.

**Table 3:** Estimated changes in self‐reported physical activity measures stratified by treatment

Physical activity assessment	Time since initiation of chemotherapy	Dox mean change (95% CI)	*P*‐value	Tras mean change (95% CI)	*P*‐value	Dox + Tras mean change (95% CI)	*P*‐value
Total activity score Median (IQR)	3 mo	−0.57 (−1.65, 0.51)	.298	0.11 (−1.82, 2.03)	.915	−1.26 (−3.21, 0.70)	.209
	6 mo	0.32 (−0.81, 1.45)	.579	0.68 (−1.86, 3.21)	.602	−1.13 (−3.52, 1.25)	.352
	12 mo	2.21 (0.72, 3.71)	.004	1.84 (−0.71, 4.39)	.157	0.19 (−2.18, 2.56)	.873
	24 mo	5.16 (2.96, 7.36)	<.001	3.76 (0.97, 6.55)	.008	2.96 (0.23, 5.69)	.034
	36 mo	6.95 (4.40, 9.50)	<.001	5.11 (1.87, 8.34)	.002	5.33 (2.04, 8.63)	.002
Moderate‐strenuous activity score Median (IQR)	3 mo	−0.12 (−1.14, 0.90)	.819	1.58 (−0.21, 3.38)	.084	−0.03 (−1.57, 1.51)	.971
	6 mo	0.72 (−0.37, 1.80)	.195	2.76 (0.37, 5.16)	.024	0.57 (−1.29, 2.42)	.550
	12 mo	2.46 (1.01, 3.92)	.001	4.14 (1.82, 6.47)	<.001	2.35 (0.58, 4.11)	.009
	24 mo	5.13 (2.96, 7.29)	<.001	6.00 (3.38, 8.62)	<.001	5.59 (3.37, 7.81)	<.001
	36 mo	6.65 (4.03, 9.27)	<.001	7.17 (3.98, 10.35)	<.001	8.11 (5.22, 11.00)	<.001
Sufficiently active, N (%)	3 mo	12.7 (10.4, 15.5)	.151	16.0 (12.3, 20.4)	.078	11.3 (7.7, 16.4)	.675
	6 mo	15.0 (11.9, 18.8)	.149	18.5 (14.5, 23.4)	.077	12.7 (8.8, 17.9)	.681
	12 mo	17.8 (14.6, 21.6)	.144	20.4 (16.3, 25.2)	.075	16.8 (12.2, 22.8)	.693
	24 mo	22.4 (18.7, 26.6)	.137	22.7 (17.2, 29.2)	.073	26.0 (19.5, 33.8)	.713
	36 mo	26.0 (21.4, 31.3)	.133	24.8 (18.0, 33.3)	.071	33.4 (25.0, 43.1)	.726

Note

Active systemic cancer therapy (not including radiation or hormone therapy) had the following average duration: Dox 5 mo; Tras 12 mo; Dox + Tras 15 mo.

Abbreviations: Dox, doxorubicin, Tras, trastuzumab.

For the outcome of moderate‐vigorous activity alone, a similar increase was seen across the treatment groups, with significant increases compared to baseline at 6 months in the Tras group and 12 months in the Dox and Dox + Tras groups (Table 3). In the overall cohort, only 12.1% of the cohort reported being sufficiently active at baseline; this increased to 26.0% at 36 months.

### 3.4 Associations between baseline covariates and changes in self‐reported physical activity during and after cancer treatment

Higher BMI and non‐Caucasian, non‐African American race were associated with small increases in total physical activity score over time (Table 2). In multivariable analyses, there were no significant associations between baseline demographics and the rate of change in the moderate‐strenuous activity score or the rate of change in the odds of being physically active (Tables S2 and S3).

### 3.5 Associations between baseline self‐reported physical activity and changes in cardiac function

Higher baseline total activity score was associated with a very modest improvement in the absolute value of LVEF (Beta 0.4%, 95% CI 0.1, 0.7 for each 10‐unit increase in total activity; Table 4). Given an activity score of 24 approximates 150 minutes a week of physical activity, this increase of 10 points approximates a change from sedentary to mild activity or from mild activity to moderate activity.^12^ There was a nonstatistically significant cardioprotective association between physical activity and the outcome of CTRCD (HR 0.83, 95% CI 0.66, 1.04, *P* = .097); with a total of 59 CTRCD events. There were no significant associations between total activity scores and longitudinal strain, E/E′ or time to abnormal diastolic function (Table 4). Similar results were seen using moderate‐strenuous activity score instead of total activity score (Tables S4 and S5).

**Table 4:** Associations between baseline total activity score and subsequent echocardiographic parameters or CTRCD

	Change in LVEF^a^		Time to subsequent CTRCD		Change in longitudinal strain^a^		Change in E/e'^a^		Time to abnormal diastolic function grade	
	Beta (95% CI)	*P*‐value	HR (95% CI)	*P*‐value	Beta (95% CI)	*P*‐value	Beta (95% CI)	*P*‐value	HR (95% CI)	*P*‐value
Baseline total activity score (per 10 unit increase)	0.36 (0.06, 0.66)	.020	0.83 (0.66, 1.04)	.097	−0.01 (−0.44, 0.41)	.956	−0.05 (−0.18, 0.08)	.433	1.08 (0.97, 1.21)	.170
Age	−0.02 (−0.07, 0.04)	.537	0.99 (0.97, 1.02)	.594	0.02 (−0.01, 0.06)	.173	−0.01 (−0.03, 0.01)	.572	1.06 (1.05, 1.08)	<.001
African American (reference non−African American)	0.10 (−1.21, 1.40)	.883	1.28 (0.68, 2.42)	.442	0.03 (−0.97, 1.02)	.957	0.84 (0.31, 1.36)	.002	1.54 (1.09, 2.18)	.015
Hypertension	−0.30 (−1.60, 1.00)	.652	2.13 (1.14, 3.96)	.017	−0.10 (−0.99, 0.79)	.827	0.20 (−0.37, 0.77)	.482	1.58 (1.12, 2.24)	.010
Hyperlipidemia	−1.61 (−2.94, −0.29)	.017	1.41 (0.76, 2.64)	.276	0.53 (−0.32, 1.38)	.221	−0.50 (−0.96, −0.04)	.034	0.99 (0.71, 1.38)	.953
BMI	0.09 (−0.00, 0.18)	.057	0.94 (0.89, 0.99)	.018	−0.01 (−0.08, 0.07)	.875	0.01 (−0.03, 0.05)	.573	1.00 (0.97, 1.02)	.863
Stage 2 (reference stage 1)	−0.10 (−1.42, 1.21)	.880	1.19 (0.57, 2.47)	.640	−0.65 (−1.62, 0.32)	.189	−0.28 (−0.87, 0.31)	.351	1.10 (0.74, 1.64)	.637
Stage 3 (reference stage 1)	0.47 (−1.08, 2.01)	.552	1.49 (0.64, 3.47)	.353	−0.47 (−1.61, 0.67)	.421	−0.22 (−0.92, 0.48)	.533	1.39 (0.88, 2.19)	.153
Stage 4 (reference stage 1)	−0.24 (−2.74, 2.27)	.853	1.02 (0.12, 8.59)	.983	−0.54 (−3.61, 2.53)	.731	−1.31 (−3.42, 0.80)	.223	3.57 (1.44, 8.83)	.006
Tras (reference Dox)	−0.04 (−1.34, 1.27)	.958	1.18 (0.55, 2.52)	.674	−0.08 (−1.04, 0.89)	.875	−0.42 (−0.96, 0.12)	.124	0.69 (0.46, 1.05)	.082
Dox + Tras (reference Dox)	−1.77 (−2.99, −0.54)	.005	2.21 (1.23, 3.99)	.008	0.22 (−0.61, 1.06)	.599	0.27 (−0.21, 0.75)	.263	1.39 (0.96, 2.01)	.084

^a^ Change models additionally adjusted for time since baseline using natural cubic spline with 3 *df*. Sample size for LVEF analysis was 327 patients, for longitudinal strain analysis was 296 patients, for E/e′ analysis was 323 patients. Number of events for CTRCD analysis was 59 and number of events for abnormal diastolic function grade was 198.

## 4 DISCUSSION

In this longitudinal, prospective cohort study, we determined the associations between self‐reported physical activity and echocardiography‐derived measures of systolic and diastolic function over an extended follow‐up time. Our principal findings are as follows: (a) self‐reported physical activity was low immediately prior to the initiation of systemic breast cancer treatment with anthracyclines and/or trastuzumab with only 12.1% of participants meeting national recommendations for sufficient physical activity; (b) self‐reported physical activity increased significantly in the 1‐2 years after breast cancer therapy; however, still, fewer than 30% of individuals were sufficiently active 3 years after initiation of cancer therapy; (c) in multivariable analyses, higher BMI, higher cancer stage and hyperlipidemia were associated with lower activity at baseline, while higher BMI and non‐Caucasian race were associated with smaller longitudinal increases over time; (d) self‐reported physical activity at baseline was very modestly associated with echocardiography‐derived measures of left ventricular systolic function.

Self‐reported physical activity was extremely low with only 12.1% of individuals meeting the 150 hours per week of moderate or strenuous physical activity that is recommended by multiple organizations including the American Cancer Society,^14^ American College of Cardiology/American Heart Association,^15^ and the American College of Sports Medicine.^16^ Moreover although physical activity increased over time, only 26.0% of patients were sufficiently active 3 years after initiation of cancer therapy. The physical activity level seen at 3 years in our cohort of breast cancer patients with median age of 50 years of age was similar to levels seen in a large cohort of women without cancer with mean age of approximately 15 years older (mean age 65 years).^17^ While participants treated with doxorubicin or doxorubicin followed by trastuzumab appeared to exhibit an initial decrease in physical activity, this was not observed in the trastuzumab treated patients who were not receiving anthracycline‐containing chemotherapy. However, by 3 years, physical activity increases were similar across treatment groups suggesting that interventions to increase physical activity may be generalizable across different cancer treatment regimens in the longer‐term. Prior studies have identified lower levels of physical activity in breast cancer patients treated with chemotherapy compared with no chemotherapy.^18^ To the best of our knowledge, our study is the first to compare changes in physical activity between different cancer therapy regimens. Given the known beneficial effects of exercise on cardiovascular and oncologic risk reduction as well as symptoms and quality of life, our findings highlight an important gap in primary and secondary prevention in an at‐risk population.^8^, ^16^, ^19^, ^20^

At baseline, higher BMI, higher cancer stage and hyperlipidemia were associated with lower physical activity. Those with a higher BMI were also less likely to demonstrate an increase in physical activity over time. Non‐Caucasian race, compared to Caucasians, was also associated with a smaller increase in physical activity over time. Our findings are consistent with prior studies that have shown that higher BMI is associated with lower self‐reported physical activity and greater reductions in physical activity during breast cancer treatment.^18^, ^21^, ^22^ The differences in association across race are particularly interesting; the subgroup of participants with smaller increases over time were noted to be non‐Caucasian and non‐African American (ie primarily Asian). However, these findings were based on overall small numbers of patients and warrant further study.

Given the known cardioprotective benefits of exercise, we hypothesized that higher baseline physical activity would be associated with cardioprotective effects on systolic and diastolic function during treatment with doxorubicin and/or trastuzumab. While there was a statistically significant association between higher baseline physical activity and a smaller reduction in LVEF with cancer treatment, the effect size was very small; there were no significant associations with longitudinal strain or measures of diastolic function and a nonstatistically significant association with decreased CTRCD risk (*P* = .097). We hypothesize several reasons for our largely neutral results despite the known cardioprotective effects of exercise. First, physical activity was very low in this cohort and thus the lack of association may be due to low levels of exposure. Second, due to concerns for reverse causation bias and recall bias, we only assessed the associations between physical activity immediately prior to chemotherapy initiation and subsequent echocardiographic changes; however, this one time point may not accurately reflect physical activity prior to diagnosis, during cancer therapy or after completion of therapy given the multiple events occurring at this time, including surgery. Third, the associations between exercise and clinical cardiac events may be seen with longer duration of follow up,^5^, ^23^ in other reports, the median time to first CV event was 5.5 years.^8^ Fourth, given the relatively low number of events meeting the criteria for CTRCD, we may be underpowered. Finally, exercise has multiple potential beneficial effects, not all of which can be quantified by LVEF. In the general population, exercise is associated with effects on vascular tone, endothelial function, skeletal muscle oxygen extraction, lipids, and inflammation. While we did not evaluate the associations between baseline activity levels and subsequent changes in measures of blood pressure or lipid levels, as this was outside the scope of this analyses, this is an area of future study.

There are limitations to this analysis that should be considered. First, the use of self‐reported physical activity may be limited by patient recall leading to misclassification, although the Godin questionnaire has been validated using accelerometer assessment in multiple cohorts, including breast cancer survivors.^12^ Second, we did not ask participants to recall their physical activity levels prior to cancer diagnosis; thus there is a possibility that the level of activity at the time of enrollment in the cohort may not represent the usual activity for that individual before diagnosis. Third, we did not collect information on potential predictors of physical activity level such as depression or socioeconomic status. Fourth, and as noted in our methods, we did not analyze the associations between changes in physical activity with subsequent changes in cardiac function, given concerns over reverse causation bias. Given the inability to rigorously determine a temporal relationship (ie lack of daily echocardiograms), we were unable to determine if a change in cardiac function preceded a change in physical activity, or vice versa. However, there was a moderate correlation between baseline physical activity and the reported physical activity at the second visit (correlation coefficient = 0.55) and baseline and reported physical activity at the third visit (correlation coefficient = 0.48).

In summary, a minority of individuals with breast cancer are meeting the recommended levels of physical activity and more than half report no moderate‐ strenuous physical activity at the time of systemic cancer treatment. While physical activity increases in the years after cancer treatment, only 1 in 4 breast cancer survivors are sufficiently active 3 years after diagnosis, suggesting that evidence‐based interventions to increase physical activity in this population are needed. We also observed interesting disparities across race that deserve further study. Our findings suggest modest associations between self‐reported physical activity and echocardiographic derived parameters of cardiac dysfunction over a median follow‐up of 2.3 years. In the context of the established cardioprotective benefits of exercise, our findings indicate an important need for larger studies to further understand the associations between physical activity and cardiac function; additional studies to elucidate the effects on the periphery which may confer cardioprotection; and a need for increased physical activity in this population of breast cancer patients.

## CONFLICT OF INTEREST

None declared.

## AUTHOR CONTRIBUTION

Jenica N. Upshaw contributed to conceptualization, methodology, visualization, writing, and editing. Rebecca A. Hubbard contributed to data curation, formal analysis, methodology, writing, and editing. Jian Hu contributed to data curation, formal analysis, writing, and editing. Justin C. Brown contributed to methodology, writing and editing. Amanda M. Smith contributed to investigation, project administration, writing, and editing. Biniyam Demissei contributed to methodology, writing, and editing. Kathryn H. Schmitz contributed to methodology, writing and editing. Bonnie Ky contributed to conceptualization, funding, methodology, project administration, visualization, supervision, writing, and editing.

## Supporting information

Supplementary Material

## Data Availability

The data that support the findings of this study are available from the corresponding author upon reasonable request.
