# Supplementary material for: Physical activity during and after breast cancer therapy and associations of baseline physical activity with changes in cardiac function by echocardiography
Source: Cancer Med. 2020 Jul 9;9(17):6122–31. doi: 10.1002/cam4.3277 (PMC7476829; doi:10.1002/cam4.3277)
Supplement: Supplementary file 1 — Supplementary Material [file CAM4-9-6122-s001.docx]

**Supplementary Material:**

**Supplemental Methods:**

Schedule of Echocardiographic assessment: Echocardiograms were obtained at baseline in all participants. For those in the Dox arm, echocardiograms were obtained after completion of paclitaxel and then annually. For those in the Tras arm, echocardiograms were obtained every 3 months during trastuzumab therapy and then annually. In the Dox+Tras arm, echocardiograms were obtained after completion of doxorubicin, every 3 months while receiving trastuzumab and then annually.

Diastolic function analysis: Mitral valve (MV) peak E- and A-wave velocities (cm/sec) were assessed using pulse-wave (PW) Doppler with a 1-3mm sample volume between the mitral valve leaflets in the apical four-chamber view. The average E/e’ was calculated as the MV peak E-wave velocity divided by the average of the septal and lateral e’. The 2016 American Society of Echocardiography and the European Association of Cardiovascular Imaging joint guidelines for the assessment of left ventricular diastolic function by echocardiography.

Supplemental Figure 1: Echocardiography and Questionnaire Protocol According to Treatment Regimen.

Supplemental Table 1: Number of Physical Activity Questionnaires by Treatment Group

| Doxorubicin  (N=360) | | Trastuzumab  (N=156) | | Doxorubicin  +Trastuzumab  (N=87) | |
| --- | --- | --- | --- | --- | --- |
| Months from Baseline | Number of questionnaires | Months from Baseline | Number of questionnaires | Months from Baseline | Number of questionnaires |
| 0 | 360 | 0 | 156 | 0 | 87 |
| 1 | 345 | 1 | 147 | 1 | 85 |
| 2 | 331 | 3 | 141 | 2 | 84 |
| 4 | 329 | 4 | 143 | 3 | 81 |
| 12 | 278 | 6 | 139 | 4 | 83 |
| 24 | 198 | 7 | 132 | 6 | 78 |
| 36 | 155 | 9 | 128 | 7 | 81 |
|  |  | 10 | 132 | 9 | 78 |
|  |  | 12 | 133 | 10 | 74 |
|  |  | 24 | 90 | 12 | 72 |
|  |  | 36 | 67 | 13 | 71 |
|  |  |  |  | 15 | 72 |
|  |  |  |  | 24 | 58 |
|  |  |  |  | 36 | 36 |

Godin Leisure-Time Exercise Questionnaire:


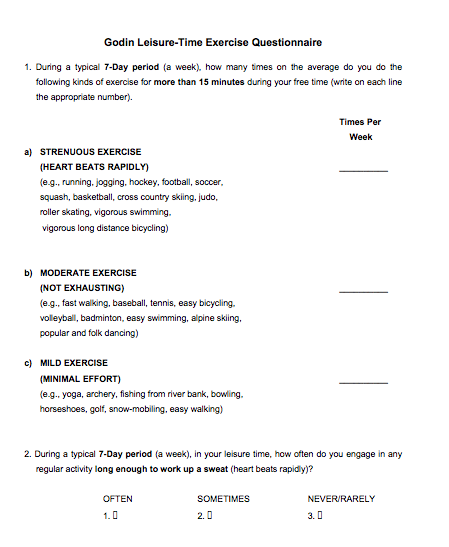


Supplemental Table 2: Associations between Baseline Clinical Characteristics and Baseline Moderate-Strenuous Activity Score and Longitudinal Rate of Change in Moderate-Strenuous Activity Score

|  | **Baseline Difference** | **95% CI** | **P-value** | **Difference in Rate of Change** | **95% CI** | **P-value** |
| --- | --- | --- | --- | --- | --- | --- |
| **Treatment (Reference Dox)** |  |  |  |  |  |  |
| **Tras** | 1.09 | (-1.18, 3.35) | 0.348 | -0.27 | (-1.40, 0.86) | 0.645 |
| **Dox+Tras** | -1.39 | (-3.59, 0.82) | 0.218 | 0.29 | (-0.63, 1.22) | 0.533 |
| **Age at baseline, quartiles, (Reference <41 years)** |  |  |  |  |  |  |
| **Quartile 2: 42-49 yrs** | -0.57 | (-3.13, 1.98) | 0.661 | -0.11 | (-1.45, 1.22) | 0.869 |
| **Quartile 3: 50-58 yrs** | 1.12 | (-1.85, 4.09) | 0.461 | -0.53 | (-1.99, 0.93) | 0.479 |
| **Quartile 4: >58 yrs** | -0.27 | (-3.20, 2.65) | 0.854 | -0.80 | (-2.21, 0.62) | 0.272 |
| **Race (Reference Caucasian)** |  |  |  |  |  |  |
| **African-American** | 0.07 | (-2.09, 2.24) | 0.948 | -0.81 | (-1.86, 0.24) | 0.132 |
| **Other** | -4.08 | (-7.28, -0.89) | 0.012 | -0.97 | (-2.64, 0.70) | 0.256 |
| **Body Mass Index** | -0.29 | (-0.43, -0.16) | <0.001 | -0.05 | (-0.13, 0.02) | 0.163 |
| **Hypertension** | -1.34 | (-3.35, 0.67) | 0.191 | -0.45 | (-1.41, 0.50) | 0.353 |
| **Smoking** | -1.00 | (-2.82, 0.81) | 0.279 | 0.52 | (-0.38, 1.41) | 0.258 |
| **Hyperlipidemia** | -1.94 | (-3.98, 0.11) | 0.064 | -0.26 | (-1.19, 0.67) | 0.586 |
| **Beta-blockers** | -1.07 | (-3.55, 1.41) | 0.397 | 0.61 | (-0.80, 2.02) | 0.397 |
| **Stage**  **(Reference 1)** |  |  |  |  |  |  |
| **2** | -2.31 | (-5.00, 0.37) | 0.092 | 0.33 | (-0.66, 1.33) | 0.512 |
| **3** | -3.89 | (-6.62, -1.16) | 0.005 | 0.32 | (-0.91, 1.56) | 0.610 |
| **4** | -3.12 | (-7.19, 0.95) | 0.133 | -1.65 | (-3.81, 0.52) | 0.136 |

Dox refers to doxorubicin, Tras to trastuzumab. Multivariable linear regression model used to estimate the association between all listed baseline demographic and clinical variables and difference in the baseline activity scores and the difference in the rate of change in the activity score during longitudinal follow-up.

Supplemental Table 3: Association between Baseline Clinical Characteristics and Odds of Being Sufficiently Active at Baseline and Multiplicative Rate of Change in Odds of Being Sufficiently Active.

|  | **Baseline Difference** | **95% CI** | **P-value** | **Difference in Rate of Change** | **95% CI** | **P-value** |
| --- | --- | --- | --- | --- | --- | --- |
| **Treatment (Reference Dox)** |  |  |  |  |  |  |
| **Tras** | 1.08 | (0.73, 1.61) | 0.695 | 0.99 | (0.86, 1.15) | 0.943 |
| **Dox+Tras** | 0.87 | (0.53, 1.44) | 0.592 | 1.11 | (0.97, 1.27) | 0.132 |
| **Age at baseline, quartiles, (Reference <41 years)** |  |  |  |  |  |  |
| **Quartile 2: 42-49 yrs** | 0.91 | (0.57, 1.45) | 0.692 | 1.02 | (0.86, 1.20) | 0.844 |
| **Quartile 3: 50-58 yrs** | 1.24 | (0.75, 2.04) | 0.402 | 0.94 | (0.79, 1.12) | 0.519 |
| **Quartile 4: >58 yrs** | 1.02 | (0.60, 1.75) | 0.938 | 0.94 | (0.79, 1.11) | 0.454 |
| **Race (Reference is Caucasian)** |  |  |  |  |  |  |
| **African-American** | 1.01 | (0.65, 1.58) | 0.959 | 0.91 | (0.79, 1.05) | 0.207 |
| **Other** | 0.38 | (0.17, 0.85) | 0.019 | 1.01 | (0.81, 1.25) | 0.957 |
| **Body Mass Index** | 0.94 | (0.91, 0.98) | 0.001 | 0.99 | (0.98, 1.01) | 0.403 |
| **Hypertension** | 0.72 | (0.45, 1.15) | 0.169 | 1.00 | (0.87, 1.16) | 0.965 |
| **Smoking** | 0.83 | (0.58, 1.18) | 0.295 | 1.09 | (0.98, 1.23) | 0.123 |
| **Hyperlipidemia** | 0.58 | (0.37, 0.90) | 0.014 | 1.02 | (0.90, 1.17) | 0.720 |
| **Beta-blockers** | 0.78 | (0.42, 1.47) | 0.450 | 1.05 | (0.82, 1.35) | 0.706 |
| **Stage**  **(Reference 1)** |  |  |  |  |  |  |
| **2** | 0.63 | (0.40, 0.98) | 0.040 | 1.07 | (0.94, 1.22) | 0.335 |
| **3** | 0.47 | (0.27, 0.82) | 0.007 | 1.09 | (0.91, 1.30) | 0.336 |
| **4** | 0.62 | (0.24, 1.56) | 0.308 | 0.44 | (0.29, 0.67) | <0.001 |

Dox refers to doxorubicin, Tras to trastuzumab. Multivariable logistic regression model used to estimate the association between all listed baseline demographic and clinical variables and difference in the baseline total activity scores and the difference in the rate of change in the odds ratio during longitudinal follow-up.

Supplemental Table 4: Associations between Baseline Moderate-Strenuous Activity Score and Subsequent Echocardiographic Parameters

|  | **Change in LVEF*** | | **Risk of Subsequent CTRCD†** | | **Change in Longitudinal Strain*** | | **Change in E/e’*** | | **Risk of Abnormal Diastolic Function Grade*** | |
| --- | --- | --- | --- | --- | --- | --- | --- | --- | --- | --- |
|  | Beta  95% CI | P-value |   HR  95% CI |   P-value | Beta  95% CI | P-value | Beta  95% CI | P-value | Beta  95% CI | P-value |
| **Baseline Moderate-Strenuous Activity Score** | 0.2  (-0.0, 0.4) | 0.116 | 0.90  (0.74, 1.22) | 0.595 | 0.0  (-0.4, 0.4) | 0.872 | -0.1  (-0.2, 0.1) | 0.378 | 1.10  (1.00, 1.22) | 0.092 |
| **Age** | -0.01  (-0.07, 0.04) | 0.647 | 0.99  (0.96, 1.02) | 0.494 | 0.02  (-0.01, 0.06) | 0.177 | -0.01  (-0.03, 0.01) | 0.554 | 1.06  (1.05, 1.08) | <0.001 |
| **African American (Ref Non African American)** | 0.19  (-1.16, 1.54) | 0.783 | 1.24  (0.66, 2.35) | 0.506 | 0.03  (-0.96, 1.02) | 0.953 | 0.82  (0.30, 1.34) | 0.002 | 1.56  (1.10, 2.22) | 0.013 |
| **Hypertension** | -0.36  (-1.66, 0.94) | 0.584 | 2.19  (1.17, 4.09) | 0.014 | -0.10  (-0.99, 0.80) | 0.833 | 0.21  (-0.36, 0.78) | 0.464 | 1.57  (1.11, 2.22) | 0.012 |
| **Hyperlipidemia** | -1.72  (-3.06, -0.39) | 0.012 | 1.51  (0.81, 2.79) | 0.194 | 0.54  (-0.31, 1.39) | 0.215 | -0.49  (-0.95, -0.02) | 0.040 | 0.98  (0.70, 1.36) | 0.884 |
| **BMI** | 0.08  (-0.01, 0.17) | 0.080 | 0.94  (0.90, 0.99) | 0.026 | -0.00  (-0.08, 0.07) | 0.911 | 0.01  (-0.03, 0.05) | 0.591 | 1.00  (0.97, 1.02) | 0.865 |
| **Stage 2**  **(Reference Stage 1)** | -0.16  (-1.49, 1.17) | 0.815 | 1.26  (0.61, 2.60) | 0.532 | -0.64  (-1.61, 0.33) | 0.198 | -0.27  (-0.86, 0.31) | 0.358 | 1.10  (0.74, 1.64) | 0.621 |
| **Stage 3**  **(Reference Stage 1)** | 0.38  (-1.18, 1.95) | 0.632 | 1.55  (0.67, 3.58) | 0.310 | -0.45  (-1.61, 0.70) | 0.443 | -0.22  (-0.91, 0.47) | 0.529 | 1.38  (0.88, 2.17) | 0.157 |
| **Stage 4**  **(Reference Stage 1)** | -0.52  (-3.02, 1.97) | 0.681 | 1.23  (0.15, 10.22) | 0.849 | -0.51  (-3.56, 2.54) | 0.741 | -1.28  (-3.39, 0.82) | 0.233 | 3.44  (1.40, 8.44) | 0.007 |
| **Tras**  **(Reference Dox)** | -0.05  (-1.36, 1.26) | 0.938 | 1.17  (0.55, 2.50) | 0.686 | -0.06  (-1.02, 0.90) | 0.902 | -0.43  (-0.97, 0.11) | 0.118 | 0.71  (0.47, 1.06) | 0.096 |
| **Dox+Tras**  **(Reference Dox)** | -1.76  (-3.00, -0.53) | 0.005 | 2.22  (1.23, 4.02) | 0.008 | 0.24  (-0.61, 1.09) | 0.583 | 0.26  (-0.22, 0.74) | 0.292 | 1.41  (0.97, 2.05) | 0.068 |

*Change models additionally adjusted for time since baseline using natural cubic spline with 3 degrees of freedom. Sample size for LVEF analysis was 327 patients, for longitudinal strain analysis was 296 patients, for E/e’ analysis was 323 patients. Number of events for CTRCD analysis was 59 and number of events for abnormal diastolic function grade was 198.

Supplemental Table 5: Associations between Baseline Sufficiently Active and Subsequent Echocardiographic Parameters

|  | **Change in LVEF*** | | **Risk of Subsequent CTRCD†** | | **Change in Longitudinal Strain*** | | **Change in E/e’*** | | **Risk of Abnormal Diastolic Function Grade*** | |
| --- | --- | --- | --- | --- | --- | --- | --- | --- | --- | --- |
|  | Beta  95% CI | P-value |   HR  95% CI |   P-value | Beta  95% CI | P-value | Beta  95% CI | P-value | Beta  95% CI | P-value |
| **Sufficiently active** | 0.56  (-0.76, 1.88) | 0.405 | 0.77  (0.33, 1.83) | 0.562 | -0.23  (-1.35, 0.89) | 0.692 | -0.33  (-0.98, 0.33) | 0.329 | 1.30  (0.81, 2.07) | 0.271 |
| **Age** | -0.01  (-0.07, 0.04) | 0.667 | 0.99  (0.96, 1.02) | 0.504 | 0.02  (-0.01, 0.06) | 0.177 | -0.01  (-0.03, 0.01) | 0.560 | 1.06  (1.05, 1.08) | <0.001 |
| **African American (Ref non African American)** | 0.17  (-1.18, 1.51) | 0.808 | 1.25  (0.66, 2.37) | 0.485 | 0.03  (-0.96, 1.02) | 0.957 | 0.84  (0.31, 1.36) | 0.002 | 1.53  (1.08, 2.17) | 0.016 |
| **Hypertension** | -0.39  (-1.70, 0.92) | 0.555 | 2.19  (1.18, 4.09) | 0.013 | -0.09  (-0.99, 0.81) | 0.844 | 0.22  (-0.35, 0.79) | 0.456 | 1.56  (1.10, 2.20) | 0.013 |
| **Hyperlipidemia** | -1.71  (-3.06, -0.37) | 0.012 | 1.49  (0.80, 2.77) | 0.208 | 0.51  (-0.35, 1.38) | 0.244 | -0.50  (-0.97, -0.04) | 0.035 | 1.00  (0.71, 1.40) | 0.985 |
| **BMI** | 0.08  (-0.01, 0.17) | 0.100 | 0.94  (0.90, 0.99) | 0.027 | -0.01  (-0.08, 0.07) | 0.856 | 0.01  (-0.03, 0.05) | 0.561 | 1.00  (0.97, 1.02) | 0.810 |
| **Stage 2**  **(Reference Stage 1)** | -0.13  (-1.45, 1.20) | 0.853 | 1.24  (0.60, 2.57) | 0.563 | -0.67  (-1.65, 0.31) | 0.178 | -0.30  (-0.89, 0.29) | 0.321 | 1.11  (0.75, 1.66) | 0.601 |
| **Stage 3**  **(Reference Stage 1)** | 0.37  (-1.18, 1.93) | 0.637 | 1.53  (0.66, 3.55) | 0.322 | -0.49  (-1.64, 0.66) | 0.402 | -0.24  (-0.94, 0.46) | 0.497 | 1.39  (0.88, 2.19) | 0.158 |
| **Stage 4**  **(Reference Stage 1)** | -0.53  (-3.03, 1.97) | 0.676 | 1.22  (0.15, 10.17) | 0.852 | -0.55  (-3.61, 2.50) | 0.722 | -1.29  (-3.38, 0.80) | 0.226 | 3.42  (1.39, 8.42) | 0.007 |
| **Tras**  **(Reference Dox)** | -0.03  (-1.34, 1.28) | 0.938 | 1.15  (0.54, 2.47) | 0.714 | -0.11  (-1.08, 0.86) | 0.824 | -0.46  (-1.00, 0.09) | 0.100 | 0.71  (0.47, 1.07) | 0.098 |
| **Dox+Tras**  **(Reference Dox)** | -1.81  (-3.04, -0.57) | 0.005 | 2.24  (1.24, 4.04) | 0.007 | 0.22  (-0.62, 1.05) | 0.609 | 0.27  (-0.21, 0.75) | 0.267 | 1.39  (0.96, 2.02) | 0.082 |

*Change models additionally adjusted for time since baseline using natural cubic spline with 3 degrees of freedom.
